# Bone Bridge Formation in Hydroxyapatite Spacers Following Cervical Laminoplasty: A Comparative Study of Spondylotic Myelopathy and Ossification of the Posterior Longitudinal Ligament

**DOI:** 10.7759/cureus.80861

**Published:** 2025-03-19

**Authors:** Hirotomo Tanaka, Yoshiyuki Takaishi, Hiroto Kajimoto, Masahiro Sugihara, Takeshi Kondoh, Takashi Sasayama

**Affiliations:** 1 Neurosurgery, Shinsuma General Hospital, Kobe, JPN; 2 Neurosurgery, Kobe University Graduate School of Medicine, Kobe, JPN

**Keywords:** bone bridge between spacers, cervical laminoplasty, hydroxyapatite spacer, long-term outcome, opll, spondylotic myelopathy

## Abstract

Introduction: Cervical laminoplasty using hydroxyapatite spacers is a surgical procedure widely performed for managing spondylotic myelopathy and ossification of the posterior longitudinal ligament (OPLL). Despite the procedure’s efficacy, the mechanisms underlying bone formation in OPLL remain poorly understood, and postoperative ectopic ossification may still occur. After laminoplasty, we compared bone bridge formation between adjacent hydroxyapatite spacers in patients with cervical spondylotic myelopathy and OPLL.

Materials and methods: A retrospective analysis was conducted on 30 patients who underwent double-door laminoplasty involving two or more consecutive laminae between 2006 and 2020. The median patient age was 62 years (42-87 years), with 21 male and nine female patients. Bone bridge formation over an extended postoperative period was evaluated and compared between spondylotic myelopathy (n=16) and OPLL groups (n=14).

Results: The median duration from laminoplasty to the last postoperative CT scan was 65 months in the spondylotic myelopathy group and 84 months in the OPLL group. No significant intergroup difference was observed between groups (p=0.45). New bone bridges were identified in five of the 16 patients with spondylotic myelopathy (31%) and nine of the 14 patients with OPLL (64%). The difference was not statistically significant (p=0.079). Throughout the observation period, all but one patient demonstrated stable neurological function.

Conclusions: The incidence of bone bridge formation between adjacent hydroxyapatite spacers did not significantly differ between patients with and without spondylotic myelopathy. Hydroxyapatite spacers in laminoplasty were safe and effective under both conditions.

## Introduction

The primary pathologies underlying cervical spinal stenosis are spondylotic myelopathy and ossification of the posterior longitudinal ligament (OPLL). Surgical intervention is the only effective treatment option for symptomatic patients presenting with myelopathy. Historically, laminectomy was the standard surgical approach; however, postoperative complications such as laminectomy membrane formation, instability, and kyphotic deformity were commonly reported. Since the 1980s, laminoplasty has emerged as the preferred surgical method for cervical spondylotic myelopathy, or OPLL, involving multiple intervertebral lesions without significant vertebral slippage, instability, or kyphotic deformity [1]. Laminoplasty can be performed using a double-door or open-door technique, which is widely utilized. While some studies have compared the postoperative outcomes of these two techniques, no definitive differences have been identified [2-4].

During laminoplasty, autologous bone harvested from the iliac crest was used as a spacer between the opened lamina. However, various artificial spacers are now available, with hydroxyapatite spacers being among the most commonly used due to their high bone-fusion rates. Although short- and medium-term complications associated with hydroxyapatite spacers, such as spacer displacement and dural laceration, have been documented [5-7], limited information is available on long-term postoperative radiological changes.

This study aimed to investigate the formation of new bone bridges between adjacent hydroxyapatite spacers, which may contribute to cervical spinal stenosis or reduced cervical mobility over an extended postoperative period. Additionally, the study compared the incidence and characteristics of bone bridge formation between patients with spondylotic myelopathy and those with OPLL.

## Materials and methods

Study design and patient characteristics

We conducted a retrospective review of patients with spondylotic myelopathy or OPLL who underwent double-door cervical laminoplasty using hydroxyapatite spacers at our institution between 2006 and 2020. The inclusion criteria were (1) a confirmed diagnosis of myelopathy caused by spondylotic myelopathy or OPLL, (2) availability of cervical spine CT scans taken preoperatively and at least two years postoperatively, and (3) age 20 years or older at the time of surgery. The exclusion criteria were (1) a history of cervical spine surgery and (2) renal dysfunction, defined as serum creatinine levels exceeding 1.2 mg/dL or being on dialysis. This study was reviewed and approved by the Institutional Review Board of Shinsuma General Hospital (approval number: 2024RIN-7). The requirement for written informed consent was waived due to the study's retrospective nature. Informed consent for the publication of the image shown in the figure was obtained from the relevant participant.

Surgical techniques and postoperative management

All patients underwent surgery under general anesthesia in the prone position with head stabilization using a Mayfield skull clamp. The same surgical procedure was employed for all cases, with the aid of surgical loupes. Double-door laminoplasty was performed on two or more contiguous laminae within the C3-C7 range. In cases where additional decompression was necessary, partial laminectomy or posterior foraminotomy of the affected nerve root was performed. The ligamentum flavum was resected laterally as much as possible to facilitate spinal canal expansion. The midline of the lamina to be expanded was opened, and the width of the expanded area was assessed using a trial spacer. A hydroxyapatite spacer of the appropriate size (large, medium, or small) was then selected and positioned. All cases utilized hydroxyapatite intraspinous spacers with 40% porosity (HOYA Technosurgical, Tokyo, Japan) to achieve spinal canal expansion. The spacers were secured to the open lamina using non-absorbable sutures. No additional implants were used in any of the procedures. Patients were instructed to avoid wearing a cervical collar postoperatively and began rehabilitation the day after surgery.

Evaluation of CT images and functional status

Cervical spine CT images obtained preoperatively and at least two years postoperatively were evaluated for all cases. The formation of new bone bridges between adjacent spacers was assessed using sagittal and coronal CT images. The earliest scan demonstrating new bone bridge formation was selected for evaluation for patients who underwent multiple CT scans more than two years post-surgery. The final CT scan obtained during the observation period was used to analyze patients without evidence of new bone bridge formation. Additionally, the bony union between the hydroxyapatite spacer and the lamina to which it was fixed was evaluated based on the Ichikawa classification [8]. Neurological function was assessed using the Neurosurgical Cervical Spine Scale (NCSS) and the Japanese Orthopedic Association (JOA) score preoperatively and at the last follow-up.

Statistical analysis

Data analysis was conducted using the statistical software package EZR (Jichi Medical University Saitama Medical Center, Tokyo, Japan), which is a graphical user interface for R (R Foundation for Statistical Computing, Vienna, Austria) [9]. The Mann-Whitney U test was used to compare the two groups, with a p-value of less than 0.05, which is considered statistically significant.

## Results

New bone bridge formation between the adjacent hydroxyapatite spacers

Thirty patients with a total of 105 hydroxyapatite spacers (56 in the spondylotic myelopathy group and 49 in the OPLL group) were included in this study. The median age of all participants was 62 years, comprising 21 males and nine females. Sixteen patients were in the spondylotic myelopathy group, with a median age of 68 years and a male-to-female ratio of 11:5, while 14 patients were in the OPLL group, with a median age of 58 years and a male-to-female ratio of 10:4. The median number of hydroxyapatite spacers used per case was 3.5 in both groups (range: spondylotic myelopathy group, 3-4; OPLL group, 2-5). The median operative times were 154.5 minutes for the spondylotic myelopathy group and 163 minutes for the OPLL group, with median intraoperative blood losses of 125 mL and 107.5 mL, respectively. No intraoperative dural injuries or major procedure-related complications were observed. Furthermore, no dislocation or fracture of the hydroxyapatite spacers was noted during the postoperative period.

Among non-cervical spine surgeries, five of the 16 patients in the spondylotic myelopathy group underwent posterior lumbar decompression or percutaneous vertebroplasty either before or after laminoplasty.

The median time from laminoplasty to postoperative cervical spine CT was 65 months in the spondylotic myelopathy group and 84 months in the OPLL group, with no significant difference (p=0.45). Formation of one or more new bone bridges between adjacent hydroxyapatite spacers was observed in five of the 16 patients in the spondylotic myelopathy group (31%) and nine of the 14 patients in the OPLL group (64%). This difference was not statistically significant (p=0.079; Table 1). On long-term postoperative cervical spine CT, the bony union between the hydroxyapatite spacer and the opened lamina was classified as D or E according to the Ichikawa classification in 55% of patients in the spondylotic myelopathy group and 69% in the OPLL group.

**Table 1:** Intergroup comparison of patient characteristics and bone bridge formation between adjacent hydroxyapatite spacers OPLL: ossification of the posterior longitudinal ligament, CT: computed tomography

Characteristics		No. (%)		p-value
		Spondylotic myelopathy group (n=16)	OPLL group (n=14)	
Age, median (range), y		68 (42-82)	58 (49-87)	0.12
Sex				0.90
	Male	11 (68)	10 (71)	
	Female	5 (32)	4 (29)	
Period from surgery to postoperative CT scan evaluated, median (range), m		65 (27-136)	84 (27-190)	0.45
Median number of spacers used per case		3.5	3.5	
Number of cases with bone bridge formation between adjacent spacers in one or more locations		5 (31)	9 (64)	0.079
Bone union between spacers and opened lamina (Ichikawa classification)				
A		2	2	
B		4	0	
C		19	13	
D		23	22	
E		8	12	

Radiological evaluation

**Figure 1:**
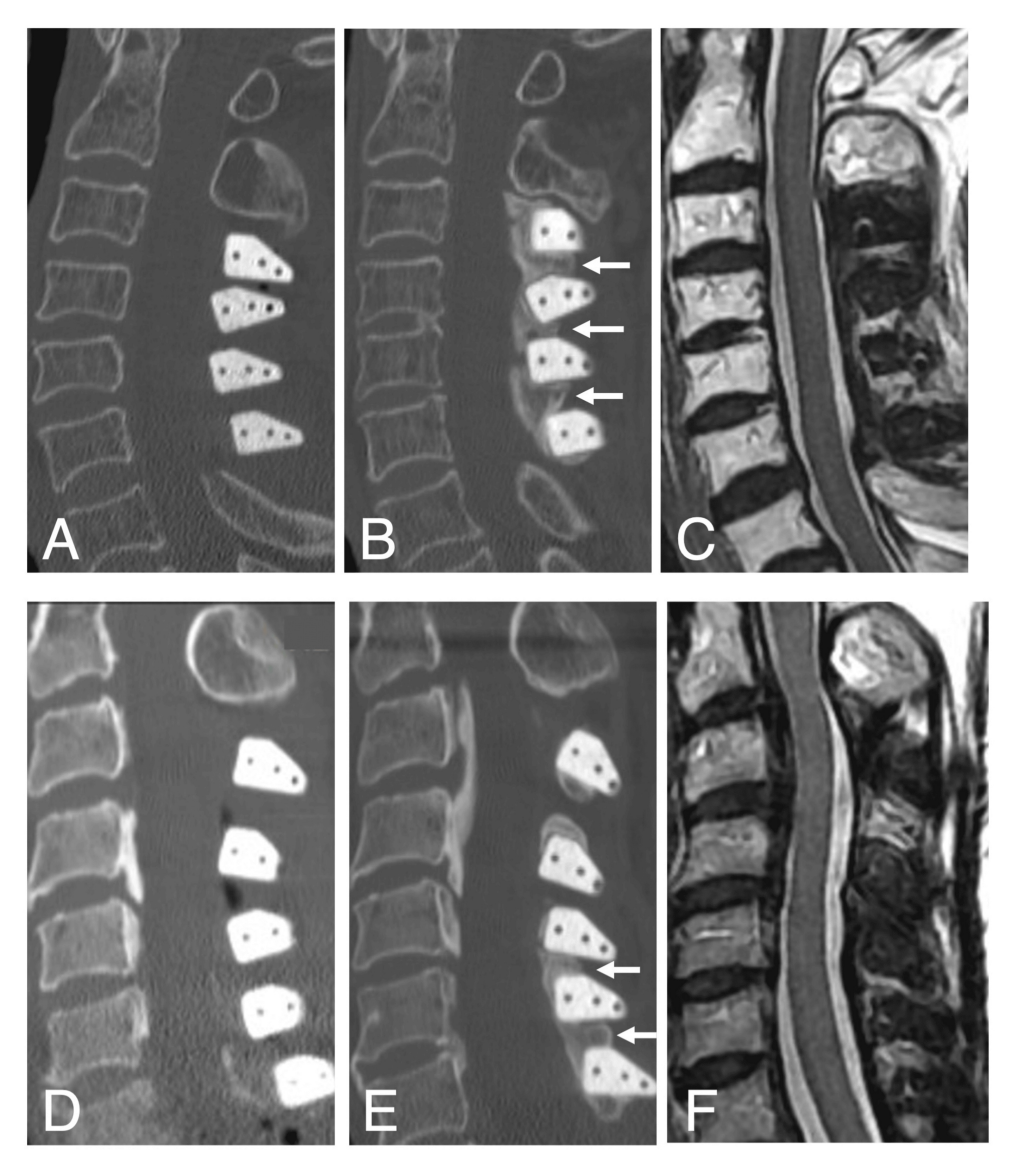
CT and MRI images of the cervical spine in cases of bone bridge formation Sagittal CT images of the cervical spine in patients with spondylotic myelopathy and OPLL immediately after surgery (A, D) and 52 and 93 months after surgery (B, E), respectively, demonstrating remarkable new bone bridge formation between adjacent hydroxyapatite spacers after the long-term postoperative period (white arrows). Sagittal MRI T2-weighted images of the same patients with spondylotic myelopathy and OPLL at 93 and 92 months after surgery, respectively (C, F), demonstrating that the subarachnoid space was well preserved. CT: computed tomography: MRI: magnetic resonance imaging, OPLL: ossification of the posterior longitudinal ligament

Preoperative cervical spine CT revealed a positive K-line in all cases, with no evidence of significant vertebral slippage or ossification/calcification of the ligamentum flavum. In the OPLL group, the types of OPLL determined by preoperative CT were as follows: seven cases of mixed type, five cases of segmental type, and two cases of continuous type. For both groups, cervical spine CT scans were performed at the discretion of the attending physician when patients reported symptoms such as neck pain or mild radiculopathy or following trauma. The bone bridges identified in this study were discovered incidentally on CT images obtained during such evaluations. Figure 1 presents typical examples of bone bridge formation observed in patients with spondylotic myelopathy and OPLL on cervical spine CT images. Only one patient in the OPLL group had evidence of spinal cord compression on cervical spine MRI more than eight years after surgery.

Preoperative cervical spine MRI was performed for all patients except one in the spondylotic myelopathy group. Among the 15 patients in the spondylotic myelopathy group and 13 patients in the OPLL group who underwent MRI, intramedullary hyperintensity within the area of spinal canal stenosis was observed on T2-weighted images. For the one patient in the spondylotic myelopathy group who could not undergo MRI due to the presence of metal implants, CT myelography was performed as an alternative diagnostic modality.

Neurofunctional outcomes

Among the 30 patients, detailed medical records related to neurological function were unavailable for three patients in the spondylotic myelopathy group and one in the OPLL group, resulting in a neurofunctional evaluation performed in 26 patients. The median observation periods for neurofunctional evaluation were 104 months for the spondylotic myelopathy group and 97 months for the OPLL group. The NCSS and JOA scores demonstrated improvement following laminoplasty in both groups (Table 2). Of the 13 patients with spondylotic myelopathy who were evaluated for neurofunctional outcomes, three had also undergone lumbar laminectomy for lumbar spinal canal stenosis, either before or after laminoplasty. Additionally, two patients in each group sustained cervical spinal cord injuries due to falls prior to undergoing surgery.

**Table 2:** Comparison of neurofunctional scores between the two groups 1: Detailed data on neurofunction were unavailable for three of 16 patients and one of 14 patients in the spondylotic myelopathy and OPLL groups, respectively. 2: Three of 13 spondylotic myelopathy patients evaluated for neurofunctional status underwent posterior lumbar decompression before or after cervical laminoplasty. NCSS: Neurosurgical Cervical Spine Scale, JOA: Japanese Orthopedic Association, OPLL: ossification of the posterior longitudinal ligament

Characteristics	Spondylotic myelopathy group (n=13)^1,2^	OPLL group (n=13)^1^	P-value
Period from surgery to postoperative neurofunctional assessment, median (range), m	104 (54-154)	97 (40-205)	0.66
NCSS, median (range)			
Preoperative	10 (5-13)	9 (5-13)	
Last follow-up	11 (7-13)	11 (6-14)	
JOA score, median (range)			
Preoperative	10 (3-15)	10 (4-16)	
Last follow-up	11 (4-16)	14 (7-16)	

**Figure 2:**
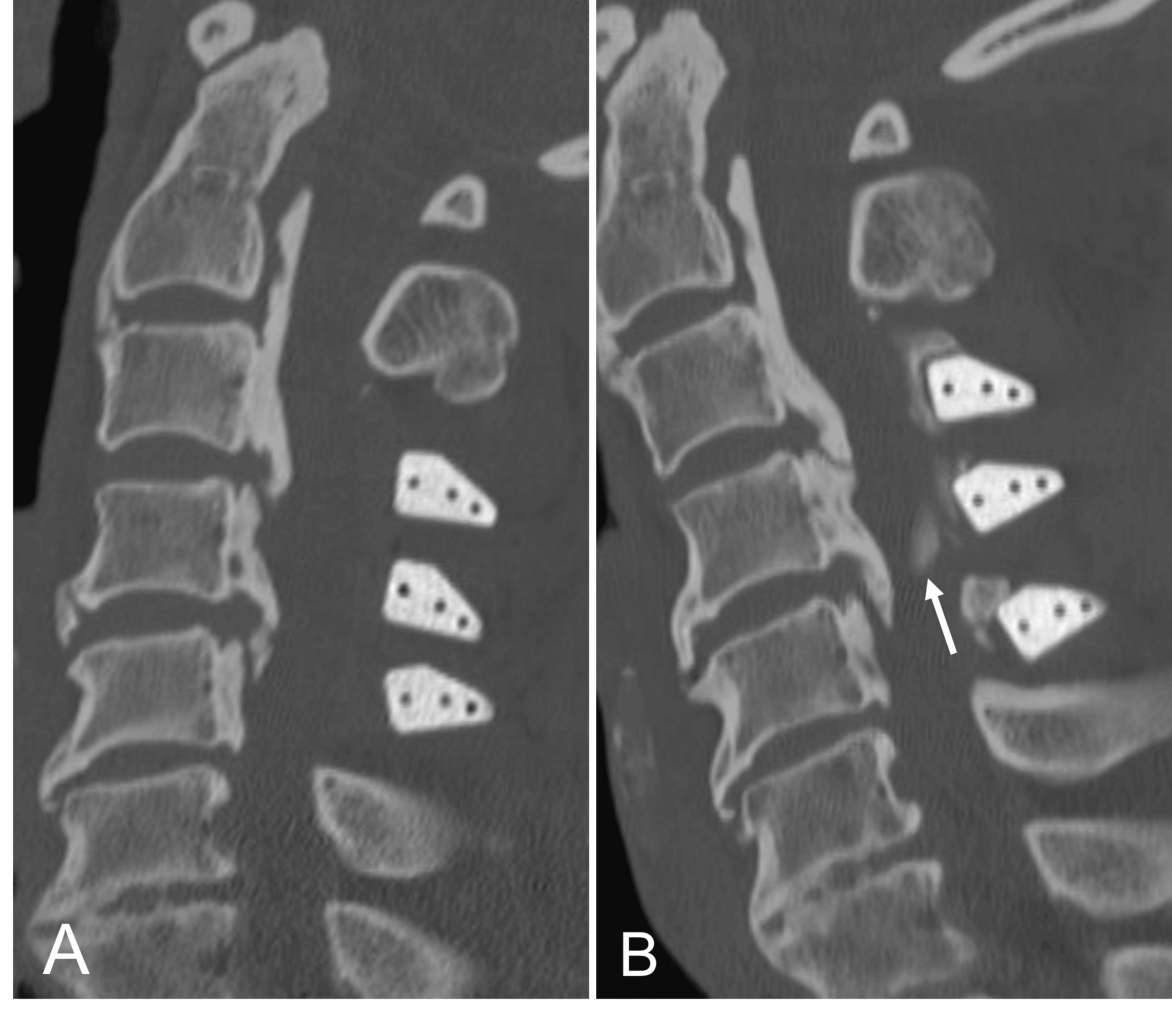
CT images of the cervical spine in the case that required reoperation Sagittal view of the cervical spine CT images immediately (A) and 102 months (B) after surgery of the OPLL patient who required reoperation showing osteophyte-like new bone formation resulting in spinal canal stenosis at the C4/5 level (white arrow). No obvious bone bridging between spacers was observed. CT: computed tomography, OPLL: ossification of the posterior longitudinal ligament

In the OPLL group, one patient underwent reoperation 102 months after laminoplasty due to spinal canal compression caused by isolated new bone formation rather than bony bridging. Preoperative CT imaging revealed no evidence of bone bridge formation between the spacers. However, new osteophyte-like thick bone formation was observed on the dorsal aspect of the dural sac at the C4/5 level, resulting in worsening myelopathy (Figure 2). Consequently, the patient underwent a posterior laminectomy and anterior decompression with fusion as a corrective surgical procedure.

## Discussion

Cervical spondylotic myelopathy is a common spinal cord disorder, with its prevalence expected to rise as the aging population grows. Most patients with spondylotic myelopathy experience a stepwise progression of myelopathy characterized by alternating periods of deterioration and stability. In contrast, OPLL is a relatively rare and intractable condition of uncertain etiology, primarily affecting the cervical spine [10]. Genetic factors play a significant role in OPLL pathogenesis, which is more frequently observed in East Asia than in other regions [11-13]. When severe spinal cord symptoms develop, conservative treatments are typically ineffective, and early surgical intervention becomes necessary.

Laminoplasty has become a key surgical procedure for treating cervical spinal stenosis with myelopathy. Since its introduction in the 1980s, this technique has consistently demonstrated favorable clinical outcomes [14]. While numerous studies have reported on short- and medium-term postoperative outcomes for cervical spinal stenosis [15-17], there is limited research on long-term radiological changes following laminoplasty. Iwasaki et al., in a study monitoring 64 OPLL patients who underwent laminoplasty for over a decade, identified cases of late neurological deterioration attributed to lumbar degenerative disease, ossification of the thoracic ligamentum flavum, and progression of OPLL [18]. Although progression of ossified lesions was observed in 70% of patients, only 3% exhibited symptom worsening, and additional cervical surgery was required in just 2% of cases.

Hydroxyapatite, polyetheretherketone, and titanium are commonly used for spacers in laminoplasty. The choice of spacer is often based on the surgeon’s preference. Our institution has performed double-door laminoplasty using hydroxyapatite spacers for over two decades. This approach allows symmetrical spinal canal decompression, with relatively straightforward fixation of hydroxyapatite spacers using non-absorbable sutures.

Hydroxyapatite, the primary inorganic component of living bone, is extensively used in orthopedic, spinal, and dental surgeries due to its excellent osteoconductivity and biocompatibility [19]. Its porosity facilitates osteoblast anchoring and proliferation, promoting bony union [20]. Studies have indicated that higher porosity (e.g., 50%) spacers facilitate early bone bonding but may be prone to complications such as breakage [21]. Iguchi et al. reported in a study of 33 patients who underwent laminoplasty using 60% microporosity hydroxyapatite spacers that over a mean observation period of 30 months, rigid bony union and peri-spacer bone formation were achieved in 61 and 91% of cases, respectively [8].

Although several studies have reported the bony union of hydroxyapatite spacers, data on excessive bone formation associated with hydroxyapatite spacers in laminoplasty are scarce. MRI is often used for long-term imaging follow-up after laminoplasty, and CT is rarely performed. This finding of bone bridging could only be noted with CT and, therefore, has probably not been reported previously.

This study of 30 cases revealed that new bone bridge formation between adjacent hydroxyapatite spacers is a common long-term occurrence following laminoplasty. Although this bone bridging does not typically result in direct compression of the dural sac, new bone formation on the posterior side of the dural sac caused neurological deterioration in one patient, necessitating revision surgery. This underscores the need for spine surgeons to be vigilant about the potential for excessive bone formation, which may lead to symptoms requiring reoperation. Furthermore, new bone bridge formation could lead to long-term complications, such as reduced intervertebral range of motion and adjacent segment disease, particularly in spondylotic myelopathy cases without anterior fixation. On the other hand, in spondylotic myelopathy cases, posterior fixation by bone bridge formation may suppress dynamic factors contributing to spinal cord damage and help prevent kyphotic deformities. Therefore, whether new bone bridge formation should be considered a clinically significant complication remains debatable, as it may confer certain protective benefits.

In comparing spondylotic myelopathy and OPLL groups, our initial hypothesis was that the frequency of new bone bridge formation would be higher in the OPLL group due to its systemic osteogenesis background [22]. However, this study found no significant difference between the two groups. The lack of statistical significance may be attributed to the small sample size and limited statistical power.

This study has several limitations, including its retrospective design and small sample size. Additionally, systemic complications and the timing of postoperative CT imaging were not accounted for, which may influence the results. Future studies should address these limitations by incorporating larger patient cohorts and conducting comparative analyses involving alternative spacer materials.

## Conclusions

Bone bridge formation between adjacent hydroxyapatite spacers rarely results in severe neurological deterioration requiring reoperation. Hydroxyapatite spacers can safely be used in laminoplasty for spondylotic myelopathy and OPLL patients. Future research should focus on accumulating larger datasets and comparing outcomes with other spacer types to better understand long-term implications.
